# Síntesis de evidencia y recomendaciones: Guía para el cuidado de pacientes adultos críticos con COVID-19 en las Américas

**DOI:** 10.26633/RPSP.2021.128

**Published:** 2021-11-03

**Authors:** Organización Panamericana de la Salud

**Affiliations:** 1 Organización Panamericana de la Salud Washington, D.C. Estados Unidos de América Organización Panamericana de la Salud, Washington, D.C., Estados Unidos de América.

**Keywords:** COVID-19, coronavirus, medicina basada en evidencia, infecciones respiratorias, unidades de cuidados intensivos, terapéutica, Américas, COVID-19, coronavirus, evidence-based medicine, respiratory tract infections, intensive care units, therapeutics, Americas, COVID-19, coronavírus, medicina baseada em evidências, infecções respiratorias, unidades de terapia intensiva, terapêutica, Américas

## Abstract

**Introducción.:**

La Organización Mundial de la Salud declaró en el 2020 la pandemia de la enfermedad por el coronavirus 2019 (COVID-19, por su sigla en inglés), causada por el coronavirus de tipo del síndrome respiratorio agudo grave (SARS-CoV-2, por su sigla en inglés) y que se ha extendido alrededor del mundo. Aproximadamente 5% de los pacientes infectados son críticos y requieren admisión a la unidad de cuidado intensivo (UCI). En estos pacientes, la COVID-19 puede estar complicada con un síndrome de dificultad respiratoria aguda, choque séptico y falla multiorgánica, que incluye la falla renal y cardíaca.

**Objetivo.:**

Sintetizar las recomendaciones incluidas en la *Guía para el cuidado de pacientes adultos críticos con COVID-19 en las Américas,* *versión* *3* publicada por la Organización Panamericana de la Salud en 2021, con el fin de orientar el manejo de pacientes adultos críticos con COVID-19 atendidos en la UCI y abordar aspectos clave de su implementación.

**Métodos.:**

Se llevó a cabo una síntesis de la guía y sus recomendaciones. Además, se realizó una búsqueda sistemática en Pubmed, Lilacs, Health Systems Evidence, Epistemonikos y literatura gris de estudios desarrollados en la Región de las Américas con el fin de identificar barreras, facilitadores y estrategias de implementación.

**Resultados.:**

Se presentan 43 recomendaciones que abordan la identificación de marcadores y factores de riesgo de mortalidad, prevención y control de infecciones, recolección de muestras, cuidado de soporte (ventilatorio y hemodinámico), tratamiento farmacológico, rehabilitación temprana, uso de estudios por imágenes, prevención de complicaciones y criterios de egreso de los pacientes críticos con COVID-19.

**Conclusiones.:**

Las recomendaciones buscan proveer el conocimiento sobre el manejo de pacientes críticos con COVID-19 y ofrecer consideraciones para su implementación en la Región.

## Abstract (en)

**Introduction.:**

In 2020, the World Health Organization declared the coronavirus disease 2019 (COVID-19) pandemic, caused by the severe acute respiratory syndrome coronavirus 2 (SARS-CoV-2), which has spread around the world. Approximately 5% of infected patients are critical cases that require admission to the intensive care unit (ICU). In these patients, COVID-19 can be complicated by acute respiratory distress syndrome, septic shock, and multi-organ failure, including kidney and heart failure.

**Objective.:**

Summarize the recommendations included in the Guidelines for Care of Critically Ill Adult Patients with COVID-19 in the Americas, version 3, published by the Pan American Health Organization in 2021 to guide the management of critically ill adult patients with COVID-19 treated in the ICU, and address key aspects of implementation of the recommendations.

**Methodology.:**

The guidelines and their recommendations were summarized. In addition, a systematic search was carried out in Pubmed, Lilacs, Health Systems Evidence, Epistemonikos, and gray literature on studies carried out in the Region of the Americas, in order to identify barriers, facilitators, and implementation strategies.

**Results.:**

Forty-three recommendations are presented that address the identification of markers and risk factors for mortality, infection prevention and control, specimen collection, supportive care (ventilatory and hemodynamic), pharmacological treatment, early rehabilitation, use of imaging studies, prevention of complications, and discharge criteria for critical patients with COVID-19.

**Conclusions.:**

The recommendations seek to provide knowledge on the management of critical patients with COVID-19 and offer considerations for implementation of the recommendations in the Region.

## RESUMO

**Introdução.:**

A Organização Mundial da Saúde declarou em 2020 a pandemia de COVID-19 (sigla em inglês), causada pelo coronavírus do tipo da Síndrome Respiratória Aguda Grave (SARS-COV-2, o vírus na sigla em inglês) e que tem se espalhado por todo o mundo. Aproximadamente 5% dos pacientes infectados ficam em estado crítico e precisam de cuidados em unidade de terapia intensiva (UTI). Nesses pacientes, a COVID-19 pode piorar em decorrência da síndrome do desconforto respiratório agudo, choque séptico e insuficiência de múltiplos órgãos, que inclui a falência renal e a insuficiência cardíaca.

**Objetivo.:**

Sintetizar as recomendações do *Guia para o cuidado de pacientes adultos em estado crítico com COVID-19 nas Américas, versão 3,* publicado pela Organização Pan-Americana da Saúde em 2021, a fim de orientar o manejo de pacientes adultos em estado crítico com COVID-19 atendidos em UTI e abordar aspectos importantes na sua implementação.

**Métodos.:**

Foi realizada uma síntese do guia e de suas recomendações. Além disso, foi feita uma busca sistemática nas bases Pubmed, Lilacs, Health Systems Evidence e Epistemonikos e na literatura cinzenta de estudos conduzidos na Região das Américas para identificar barreiras, facilitadores e estratégias de implementação.

**Resultados.:**

São apresentadas 43 recomendações que abordam a identificação de marcadores e fatores de risco de mortalidade, prevenção e controle de infecções, coleta de amostras, cuidados de suporte (ventilatório e hemodinâmico), tratamento farmacológico, reabilitação prematura, uso de estudos por imagens, prevenção de complicações e critérios para a alta de pacientes em estado crítico com COVID-19.

**Conclusões.:**

As recomendações buscam disponibilizar conhecimento sobre o manejo de pacientes em estado crítico com COVID-19 e oferecer considerações para sua implementação na Região.

La Organización Mundial de la Salud (OMS) declaró en el año 2020 la pandemia global de la infección causada por el coronavirus de tipo causante del síndrome respiratorio agudo grave (SARS-CoV-2, por su sigla en inglés), la cual comenzó en China y se ha extendido alrededor del mundo (1). La enfermedad por el coronavirus 2019 (COVID-19, por su sigla en inglés) es una infección del tracto respiratorio que en la mayoría de las personas se presenta como una enfermedad leve o moderada o sin complicaciones (pacientes asintomáticos). Aproximadamente 14% de los pacientes infectados desarrolla la forma grave de la enfermedad, que requiere hospitalización y soporte respiratorio, y 5% son pacientes críticos que requieren admisión en la unidad de cuidado intensivo (2,3). En estos pacientes, la COVID-19 puede estar complicada con un síndrome de dificultad respiratoria aguda, choque séptico y falla multiorgánica, que incluye la falla renal y cardíaca (4).

Las tasas de letalidad de los pacientes críticos con COVID-19 más elevadas se observaron en pacientes mayores de 60 años y pacientes con enfermedades crónicas como diabetes, hipertensión, enfermedades debilitantes del sistema inmunitario, obesidad y cáncer (5). Una gran cantidad de ensayos clínicos se iniciaron buscando evaluar las intervenciones que mejorarán los síntomas y la sobrevida de los pacientes críticos. Además, se comenzó un alto número de ensayos clínicos y se establecieron varias alianzas internacionales para identificar los tratamientos más efectivos y seguros. Entre las alianzas estratégicas, se encuentra la iniciativa de la OMS con los ensayos Solidaridad y RECOVERY, que buscan identificar cuáles monoterapias o terapias combinadas, dentro de un grupo variado de intervenciones terapéuticas, son efectivas y seguras para los pacientes con COVID-19 (6,7). Dado que es una enfermedad emergente que afecta a todos los países de la Región y aún no se conoce un tratamiento efectivo y seguro, la Organización panamericana de la Salud (OPS) actualizó su guía informada en la evidencia con el objetivo de orientar a los equipos de salud que atienden a los pacientes críticos con COVID-19, así como a los tomadores de decisiones de América Latina. La guía se estará actualizando de forma periódica a medida que se publique evidencia nueva.

El objetivo de este trabajo es presentar una síntesis de la evidencia de las recomendaciones incluidas en la *Guía para el cuidado de pacientes adultos críticos con COVID-19 en las Américas, versión 3* (8), una guía de práctica publicada por la OPS en el 2021, y aspectos clave de su implementación.

## MÉTODOS

### Objetivos y población diana considerada en la guía

La *Guía para el cuidado de pacientes adultos críticos con COVID-19 en las Américas, versión 3* se desarrolló con el objetivo de proveer recomendaciones para el manejo de pacientes adultos críticos con COVID-19 atendidos en la UCI (8). La población diana está constituida por pacientes adultos críticos con sospecha diagnóstica o confirmados con COVID-19.

De acuerdo con la OMS, se define como caso complicado al paciente que necesita soporte ventilatorio, vigilancia y manejo en la UCI y que presente las siguientes características (4): (a) cociente entre la fracción inspirada de oxígeno (FiO_2_) y la presión de oxígeno (PO_2_) ≤250; (b) radiografía de tórax con infiltrado bilateral en parches; (c) frecuencia respiratoria ≥30 respiraciones por minuto o saturación de oxígeno (satO_2_) ≤90%; y (d) presencia de síndrome de respuesta inflamatoria sistémica (SRIS), sepsis o choque séptico (8).

### Metodología de elaboración de la guía

La guía siguió los métodos de actualización rápida de los perfiles de evidencia GRADE (por su sigla en inglés) propuestos por la OPS y la OMS (9). Se conformó un grupo desarrollador multidisciplinario compuesto por expertos en medicina crítica, medicina de urgencias, infectología, anestesiología, pediatría, neumología, epidemiología y salud pública. Se realizó un proceso de selección de las preguntas a actualizar y un proceso de priorización de las preguntas a incluir. Se realizó una búsqueda sistemática de la literatura a mayo del 2021; después del proceso de selección de la evidencia se crearon y actualizaron los perfiles de evidencia GRADE (del inglés *Grading of Recommendations Assessment, Development and Evaluation*). Posteriormente, se realizó un panel en modalidad virtual con expertos iberoamericanos para formular las recomendaciones, con base en el contexto de implementación regional. Todos los miembros del grupo desarrollador firmaron un formulario de conflicto de intereses, los cuales fueron analizados por la coordinación de la guía. No se encontraron conflictos que afectaran la guía. La guía fue elaborada siguiendo la metodología GRADE, que permite formular recomendaciones considerando la calidad de la evidencia, el balance entre los riesgos y beneficios, los valores y preferencias de los pacientes, la aplicabilidad, los costos y el contexto de implementación de forma general (10). El enfoque DECIDE (11) (del inglés *Decisions and Practice based on Evidence*) se utilizó para orientar las recomendaciones basado en la calidad de la evidencia, el efecto de las intervenciones, los recursos, la equidad, la aceptabilidad y la factibilidad (11). Los detalles metodológicos y la evidencia que apoya las recomendaciones están disponibles en la guía.

### Alcance y usuarios de la guía

Esta guía de práctica clínica provee recomendaciones informadas por la evidencia para la identificación de marcadores y factores de riesgo de mortalidad de los pacientes críticos, la prevención y control de infecciones, la recolección de muestras, el cuidado de soporte (ventilatorio y hemodinámico), el tratamiento farmacológico, la rehabilitación temprana, el uso de estudios por imágenes, la prevención de complicaciones y los criterios de egreso (8).

Las recomendaciones están dirigidas a todo el personal de salud que atiende a los pacientes en el servicio de urgencias y de emergencias y la UCI (médicos especialistas en medicina de urgencias, neumología, medicina intensiva, medicina interna, anestesiología, infectología, terapistas respiratorios, terapistas físicos, enfermeras y químicos farmacéuticos). La guía está elaborada para su uso por tomadores de decisiones y miembros de entidades gubernamentales relacionados con el manejo de pacientes con COVID-19 en las UCI de la Región de las Américas. Esta guía no incluye aspectos relacionados con nutrición y manejo de complicaciones (8).

### Cómo usar la guía

Para cada pregunta clínica se presenta un grupo de recomendaciones y buenas prácticas para el manejo de pacientes críticos con COVID-19. En los cuadros 1 y 2 se presentan el nivel de calidad de la evidencia y la fuerza de la recomendación según el sistema GRADE, respectivamente.

### Metodología de desarrollo de la síntesis de evidencia y las recomendaciones

La *Guía para el cuidado de pacientes adultos críticos con COVID-19 en las Américas* (8) aborda el manejo de pacientes críticos con COVID-19, una prioridad en todo el mundo. La información de la guía relacionada con la metodología, el alcance, los objetivos, el resumen de las recomendaciones y la calidad de la evidencia se sintetizó mediante el uso de un formato predeterminado. Se utilizó la estrategia de búsqueda de la guía y filtros para identificar estudios sobre consideraciones para la implementación (11), se realizaron búsquedas de revisiones sistemáticas que abordaran aspectos de implementación (barreras, facilitadores, estrategias de implementación e indicadores). La estrategia de búsqueda incluyó los términos *adoption, uptake, utilization; taken implementation, dissemination, evidence-based treatment* y *barriers*. La búsqueda se realizó en Pubmed, Lilacs, Health Systems Evidence y Epistemonikos hasta mayo del 2021. Asimismo, se revisaron los estudios primarios y los informes técnicos desarrollados en la Región de las Américas; también se incluyeron las guías regionales y otros documentos de la OPS. No se realizó evaluación de la calidad de la evidencia incluida. Se seleccionaron revisiones sistemáticas y estudios primarios con el objetivo de identificar las consideraciones de implementación de las recomendaciones de la guía. Estas se organizaron de acuerdo con el tipo de barrera (factores humanos, preferencia de los pacientes, conocimiento de la guía, recursos y acceso). Para las barreras identificadas se seleccionaron los facilitadores y estrategias de implementación más efectivas, según el contexto de la Región. A partir de la literatura seleccionada, se identificaron y elaboraron indicadores de proceso y de resultado de implementación de la guía. Por último, un grupo interdisciplinario de metodólogos y expertos temáticos de la OPS revisaron los aspectos relacionados con la implementación.

## RESULTADOS Y DISCUSIÓN

En el cuadro 3 se presentan las recomendaciones y los puntos de buena práctica que brindan orientación para el cuidado de pacientes con COVID-19 (8). Para cada pregunta clínica, se presenta el proceso de toma de decisiones para formular las recomendaciones de acuerdo con el enfoque GRADE. Estas recomendaciones están sujetas a revisión a medida que se disponga de nueva evidencia.

## IMPLEMENTACIÓN

Se recomienda que los siguientes actores apoyen la implementación de las recomendaciones: ministerios de salud y otras entidades gubernamentales, instituciones universitarias, asociaciones científicas, personal administrativo de instituciones que brindan atención en unidades cuidado intensivo, y actores clave de los sistemas de salud de cada país.

Dentro del proceso de implementación, es determinante identificar las posibles barreras, facilitadores y las estrategias para mejorar la utilización de la guía. En el cuadro 4 se presentan algunos de estos elementos que pueden ser consideradas por los países (13–19).

**FIGURA 1.**
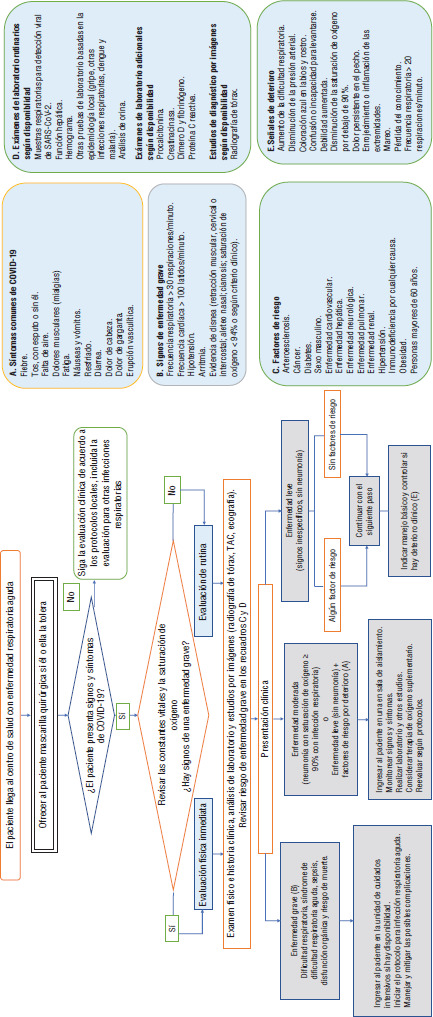
Flujograma de gestión clínica en el contexto de la COVID-19

Como estrategia de implementación de la guía, se crearon algoritmos de manejo que pueden utilizarse para el manejo de pacientes críticos con COVID-19, los cuales se presentan a continuación (figuras 1 a 3).

En el cuadro 5 se muestran los indicadores de proceso y resultado en la implementación de la guía para el cuidado de pacientes críticos con COVID-19.

**CUADRO 1.** Nivel de calidad de la evidencia según el sistema GRADE (9)

**Nivel de evidencia**	**Significado**
**Alta ⊕⊕⊕⊕**	Es muy poco probable que nuevos estudios cambien la confianza que se tiene en el resultado estimado.
**Moderada ⊕⊕⊕◯**	Es probable que nuevos estudios tengan un impacto importante en la confianza que se tiene en el resultado estimado y que estos puedan modificar el resultado.
**Baja ⊕⊕◯◯**	Es muy probable que nuevos estudios tengan un impacto importante en la confianza que se tiene en el resultado estimado y que estos puedan modificar el resultado.
**Muy baja ⊕◯◯◯**	Cualquier resultado estimado es muy incierto.

**CUADRO 2.** Fuerza de la recomendación y su significado según el sistema GRADE (9)

**Fuerza de la recomendación**	**Significado**
**Fuerte**	Debe realizarse. Es poco probable que nueva evidencia modifique la recomendación. **SE RECOMIENDA HACERLO**
**Condicional**	Podría realizarse. Nueva evidencia podría modificar la recomendación. **SE SUGIERE HACERLO**
**Fuerte en contra**	No debe realizarse. Es poco probable que nueva evidencia modifique la recomendación. **SE RECOMIENDA NO HACERLO**
**Condicional en contra**	Puede no realizarse. Nueva evidencia podría modificar la recomendación. **SE SUGIERE NO HACERLO**
**√**	Punto de buena práctica

**CUADRO 3.** Cuarenta y tres recomendaciones y veintidós puntos de buena práctica sobre el cuidado de pacientes adultos críticos con COVID-19 en las Américas

**Pregunta 1. ¿Cuáles son los factores y marcadores pronósticos de mortalidad y progresión de la enfermedad de los pacientes críticos con COVID-19?**	
**N.°**	**Recomendación**
1	Se recomienda tener en cuenta para el manejo clínico de los pacientes los siguientes factores de riesgo para la progresión de la COVID-19: edad avanzada, hipertensión, obesidad, diabetes, enfermedad cardiovascular, enfermedad pulmonar crónica (p. ej., enfermedad obstructiva crónica y asma), enfermedad renal crónica, enfermedad hepática crónica, enfermedad cerebrovascular, trombocitopenia, fumador activo, embarazo, cáncer y enfermedades que causan inmunodeficiencia. **Recomendación fuerte. Calidad de la evidencia: moderada y baja**
2	Se sugiere monitorizar, según su disponibilidad y el criterio clínico, los siguientes marcadores que han sido asociados con una mayor mortalidad en pacientes críticos con COVID-19: conteo elevado de leucocitos, deshidrogenasa láctica, proteína C reactiva, ferritina, fibrinógeno, creatinina, urea, troponina cardíaca y dímero D; así como la disminución de los niveles de albúmina y el conteo de plaquetas (marcadores relacionados con infecciones secundarias). Si se encuentra disponible, se sugiere también monitorizar la interleucina-6. **Recomendación condicional. Calidad de la evidencia: moderada y baja**
√	Se recomienda, en los pacientes críticos con COVID-19, monitorizar signos y síntomas que sugieren tromboembolismo venoso o arterial (como infarto), trombosis venosa profunda, embolismo pulmonar o síndrome coronario agudo, y proceder de acuerdo con los protocolos institucionales. **Punto de buena práctica**

**Pregunta 2. ¿Cuál es la estrategia de triaje que se debe utilizar para los pacientes críticos con COVID-19?**	
**N.°**	**Recomendación**
√	Se recomienda que se implementen protocolos institucionales para el triaje de los pacientes con sospecha diagnóstica o confirmados con COVID-19, con el fin de clasificar de forma adecuada a los pacientes que requieran manejo en una unidad de cuidados intensivos. Se deben evaluar la duración y la gravedad de los síntomas, los hallazgos de los estudios por imágenes (radiografía, tomografía computarizada o ecografía de pulmón, según su disponibilidad), el origen de infiltrados pulmonares, las necesidades de oxigenación, la disfunción de órganos vitales, la sepsis y el choque séptico para identificar a los pacientes críticos con COVID-19. La OPS cuenta con un algoritmo de manejo de pacientes con sospecha de infección por COVID-19 en el primer nivel de atención y en zonas remotas de la Región de las Américas (12). **Punto de buena práctica**

**Pregunta 3. ¿Cuál es la efectividad y seguridad de las intervenciones para prevenir la infección de los profesionales de la salud que atienden a los pacientes con COVID-19?**	
**N.°**	**Recomendación**
√	Para los trabajadores de la salud en contacto con pacientes con COVID-19 que realizan procedimientos que generan aerosoles* en la UCI o se encuentran en una unidad en la que se realizan estos procedimientos sin ventilación adecuada o un sistema independiente de presión negativa, se recomienda usar máscaras de respiración (mascarillas respiratorias N-95, FFP2 o equivalentes), en lugar de mascarillas quirúrgicas, además de otros equipos de protección personal (guantes, bata y protección para los ojos como caretas protectoras o gafas de seguridad). *Entre los procedimientos que generan aerosoles y se llevan a cabo en la UCI, se incluyen los siguientes: intubación endotraqueal, broncoscopia, aspiración abierta, tratamiento nebulizado, ventilación manual previa a la intubación endotraqueal, pronación física del paciente, desconexión del paciente del ventilador, ventilación no invasiva con presión positiva, traqueotomía y reanimación cardiopulmonar. **Punto de buena práctica**
√	Se recomienda que los procedimientos que generan aerosoles en pacientes con COVID-19 en la UCI se realicen en áreas designadas para tal propósito y cuenten con las mejores medidas disponibles para limitar la contaminación de otros pacientes o trabajadores de la salud. Si no existe disponibilidad de un cuarto con presión negativa se sugiere designar un área con ventilación natural en todas las zonas de atención de los pacientes. **Punto de buena práctica**
√	Para la ventilación natural, se recomiendan las siguientes tasas de ventilación mínima media por hora: 160 L/s/paciente (tasa de ventilación media por hora) para las salas de prevención de la transmisión aérea (con un mínimo de 80 L/s/paciente). Cuando en situaciones de urgencia u otro tipo se atienda a los pacientes en los pasillos, las tasas de ventilación deben ser las mismas que las exigidas para las salas de prevención de la transmisión aérea. Cuando la ventilación natural no es suficiente para satisfacer las exigencias recomendadas de ventilación, se recurrirá a otros sistemas de ventilación, como los de ventilación natural híbrida (mixta) y si tampoco es suficiente, se utilizará la ventilación mecánica. **Punto de buena práctica**
3	Para los trabajadores de la salud que brindan atención a pacientes con COVID-19 sin ventilación mecánica en UCI, se sugiere usar mascarillas quirúrgicas en lugar de mascarillas respiratorias, además de otros equipos de protección personal. **Recomendación condicional. Calidad de la evidencia: baja**
4	Para los trabajadores de la salud que realizan procedimientos que no generan aerosoles en pacientes con COVID-19 y ventilación mecánica (circuito cerrado), se sugiere el uso de máscaras quirúrgicas en lugar de las máscaras respiratorias, además de otros equipos de protección personal. **Recomendación condicional. Calidad de la evidencia: baja**
5	Para los trabajadores de la salud que realizan intubación endotraqueal en pacientes con COVID-19, se sugiere usar videolaringoscopio o laringoscopia directa, según la disponibilidad. **Recomendación condicional. Calidad de la evidencia: baja**
√	Para los trabajadores de la salud que realizan intubación endotraqueal en pacientes con COVID-19, se recomienda que la intubación sea realizada por un profesional de la salud experimentado en el manejo de las vías aéreas siguiendo los protocolos institucionales, con el fin de minimizar el número de intentos y el riesgo de transmisión. **Punto de buena práctica**

**Pregunta 4. ¿Cómo debe realizarse la recolección de muestras para el diagnóstico de COVID-19 en pacientes con necesidad de intubación y ventilación mecánica?**	
**N.°**	**Recomendación**
6	En pacientes adultos con sospecha de COVID-19 con necesidad de intubación y ventilación mecánica: Se sugiere llevar a cabo las pruebas de diagnóstico con muestras extraídas de las vías respiratorias inferiores (al momento de intubar o lo más cercano posible), en lugar de muestras extraídas de las vías respiratorias superiores (muestras nasofaríngeas u orofaríngeas). En el caso de las muestras de las vías respiratorias inferiores, se sugiere realizar preferiblemente un aspirado endotraqueal sobre el lavado bronquial o lavado broncoalveolar. **Recomendación condicional. Calidad de la evidencia: baja**
√	La rápida recolección y diagnóstico de las muestras de los pacientes con sospecha de COVID-19 debe ser una prioridad y debe ser realizada por profesionales expertos, siguiendo las recomendaciones de bioseguridad. Se recomienda realizar la validación institucional del procedimiento de laboratorio para el aspirado endotraqueal con el fin de evitar falsos negativos. Se deben realizar pruebas extensivas según la necesidad con el fin de confirmar el SARS-CoV-2 y las posibles coinfecciones. Se deben implementar las guías institucionales acerca de la obtención del consentimiento informado para la recolección de muestras, diagnóstico y futuras investigaciones. **Punto de buena práctica**
√	Se sugiere realizar pruebas para el diagnóstico diferencial con otras patologías (por ejemplo, la influenza, la malaria, el dengue) según las características clínicas y la epidemiologia local. **Punto de buena práctica**

**Pregunta 5. ¿Cuál es la efectividad y seguridad de las intervenciones para el soporte ventilatorio de los pacientes críticos con COVID-19?**	
**N.°**	**Recomendación**
7	En los pacientes adultos con COVID-19 con síndrome de insuficiencia respiratoria aguda (SIRA) y distrés respiratorio, hipoxemia o choque (sin intubación o ventilación mecánica), se recomienda utilizar de inmediato oxígeno suplementario hasta alcanzar SpO_2_ ≥94%. **Recomendación fuerte. Calidad de la evidencia: moderada**
8	En pacientes adultos con COVID-19 e insuficiencia respiratoria hipoxémica aguda con suplemento de oxígeno, se recomienda que la SpO_2_ no sea mayor que 96%. **Recomendación fuerte. Calidad de la evidencia: moderada**
9	En pacientes adultos con COVID-19 e insuficiencia respiratoria hipoxémica aguda con necesidad de oxígeno suplementario, se sugiere utilizar ventilación no invasiva con interfase u oxígeno nasal de alto flujo, de acuerdo con su disponibilidad, con el fin de reducir la mortalidad y la probabilidad de intubación. **Recomendación condicional. Calidad de la evidencia: muy baja**
√	En pacientes con dificultad respiratoria que presentan falla respiratoria hipoxémica aguda progresiva que no responden a la terapia de oxígeno vía máscara (tasa de flujo de 10-15 L/min que corresponde al flujo mínimo para mantener la bolsa de inflación; con FiO_2_ de 0,60-0,95), se recomienda que se les provea ventilación mecánica no invasiva (VMNI) o cánula nasal de alto flujo (CNAF) o, en su defecto, ventilación mecánica invasiva. **Punto de buena práctica**
√	El uso de oxigenoterapia con CNAF y VMNI debe restringirse a unidades donde únicamente se hospitalicen pacientes con sospecha o confirmados con COVID-19 si el ambiente tiene ventilación adecuada o presión negativa y si todo el personal en el área usa de forma correcta las medidas de protección contra aerosoles. Si esto no es posible, se prefiere la ventilación mecánica con intubación orotraqueal. **Punto de buena práctica**
10	En pacientes adultos en ventilación mecánica y con SIRA, se recomienda utilizar volúmenes corrientes bajos (4 a 8 mL/kg de peso corporal predicho) y mantener presiones meseta (*plateau*) por debajo de 30 cm H_2_O. Se requiere aplicar sedación profunda a los pacientes para lograr las metas propuestas. **Recomendación fuerte. Calidad de la evidencia: moderada**
11	En pacientes adultos en ventilación mecánica y con SIRA, se sugiere aplicar una estrategia conservadora de presión positiva al final de la espiración (PEEP) con el fin de evitar el barotrauma.* *En una estrategia con niveles altos de PEEP, el personal médico debe vigilar a los pacientes que no respondan a niveles más altos de PEEP por el riesgo de barotrauma. **Recomendación condicional. Calidad de la evidencia: baja**
12	En pacientes adultos bajo ventilación mecánica y SIRA, se recomienda utilizar una estrategia conservadora de administración de líquidos en lugar de una estrategia liberal. **Recomendación fuerte. Calidad de la evidencia: baja**
13	En pacientes adultos bajo ventilación mecánica y SIRA moderado o grave, se sugiere utilizar ventilación en posición prona durante 12 a 16 horas, en lugar de ventilación sin posición prona. **Recomendación condicional. Calidad de la evidencia: moderada**
14	En pacientes adultos sin ventilación mecánica con falla respiratoria hipoxémica, se sugiere utilizar ventilación en posición prona vigil, según la tolerancia y la respuesta de cada paciente. **Recomendación condicional. Calidad de la evidencia: baja**
√	Se debe considerar la posición prona en pacientes sedados con ventilación mecánica si presentan PEEP >10 cm H_2_O y el cociente PaO_2_/FiO_2_ <150. La posición prona requiere suficientes recursos humanos con experiencia para ser realizada de forma estandarizada y segura.
√	No se recomienda usar la posición prona en pacientes con inestabilidad hemodinámica, aumento no monitorizado de la presión intracraneana o con inestabilidad de la columna.
15	En pacientes adultos bajo ventilación mecánica y SIRA moderado o grave con altos requerimientos de ventilación mecánica: Se sugiere usar bloqueadores neuromusculares en bolos intermitentes en lugar de infusión continua para facilitar la ventilación con estrategias de protección pulmonar. En caso de asincronía ventilatoria persistente, necesidad de sedación profunda, ventilación en posición prona o persistencia de presiones *plateau* altas, se sugiere utilizar una infusión continua de bloqueadores neuromusculares durante un máximo de 48 horas. **Recomendación condicional. Calidad de la evidencia: baja**
16	En pacientes adultos bajo ventilación mecánica y SIRA, no se recomienda utilizar el óxido nítrico inhalado. **Recomendación fuerte. Calidad de la evidencia: baja**
17	En pacientes adultos en ventilación mecánica con hipoxemia refractaria a otras medidas, pese a la optimización de la ventilación, se recomienda aplicar maniobras de reclutamiento y no se recomienda usar la PEEP incremental (aumentos graduales de la PEEP). **Recomendación fuerte. Calidad de la evidencia: moderada**
18	En pacientes adultos con COVID-19 con o sin SIRA o insuficiencia respiratoria hipoxémica aguda con necesidad de oxígeno suplementario, se sugiere que se utilice la posición prona vigil por al menos 3 horas. No se debe mantener si el paciente refiere que no está cómodo o la oxigenación no mejora, esto se evalúa en los primeros 15 minutos de iniciar la posición prona vigil. **Recomendación condicional. Calidad de la evidencia: muy baja**
19	En pacientes adultos que producen o retienen secreciones o presentan tos débil, se sugiere utilizar técnicas de eliminación de secreciones (p. ej., drenado postural o maniobras de aceleración de flujo respiratorio) que contribuyen a la limpieza de las vías aéreas y aumentan la seguridad de los profesionales de salud. No se deben usar dispositivos mecánicos. **Recomendación condicional. Calidad de la evidencia: muy baja**
√	Se recomienda evitar desconectar al paciente del ventilador, dada la pérdida de PEEP, el riesgo de atelectasia y el mayor riesgo de contagio para los profesionales de la salud que atienden a los pacientes. **Punto de buena práctica**
20	Se sugiere no retardar la intubación endotraqueal en pacientes con oxígeno de flujo nasal alto (HFNO) o ventilación no invasiva (VNI) que experimentan empeoramiento de su condición o presentan el cociente PaO_2_/FiO_2_ ≤150 mmHg en un período corto de tiempo (1-2 horas). **Recomendación condicional. Calidad de la evidencia: muy baja**
21	Si se encuentra disponible, se sugiere aplicar oxigenación por membrana extracorpórea (OMEC) o remitir al paciente a un centro de OMEC en los siguientes casos críticos con COVID-19 y SIRA grave: Pacientes en ventilación mecánica con hipoxemia refractaria, quienes no responden a las alternativas terapéuticas recomendadas (optimización de la ventilación, el uso de tratamientos de rescate y la ventilación mecánica en posición prona). Se excluyen de utilizar OMEC los siguientes pacientes: Pacientes con enfermedad terminal, daño al sistema nervioso central, o que declaran el deseo de no ser reanimados o recibir OMEC. Pacientes con comorbilidades graves. Pacientes mayores de 65 años. Pacientes que han estado en ventilación mecánica por más de 7 días. **Recomendación condicional. Calidad de la evidencia: muy baja**

**Pregunta 6. ¿Cuál es la efectividad y seguridad de las intervenciones para el soporte hemodinámico de los pacientes críticos con COVID-19?**	
**N.°**	**Recomendación**
22	En la reanimación aguda de pacientes adultos con COVID-19 y en estado de choque, se sugiere aplicar una estrategia conservadora de administración de líquidos en lugar de una estrategia liberal. **Recomendación condicional. Calidad de la evidencia: muy baja**
23	En pacientes adultos con COVID-19 y en estado de choque, se sugiere utilizar alguno de los diversos parámetros dinámicos para la evaluación de la respuesta a la administración de líquidos. Entre estos, pueden ser útiles: la variación de volumen sistólico, la variación de la presión del pulso, la temperatura cutánea, el tiempo de llenado capilar, o los niveles de lactato. **Recomendación condicional. Calidad de la evidencia: baja**
24	En la reanimación aguda de pacientes adultos con COVID-19 y en estado de choque, se recomienda administrar 250 a 500 mL de volumen con cristaloides en lugar de coloides. Los cristaloides incluyen solución salina normal y Ringer lactato. **Recomendación fuerte. Calidad de la evidencia: baja**
25	En la reanimación aguda de pacientes adultos con COVID-19 y en estado de choque, se sugiere administrar soluciones cristaloides balanceadas en lugar de cristaloides no balanceadas donde se encuentren disponibles. Las soluciones balanceadas incluyen lactato de Ringer u otras soluciones multielectrolíticas. **Recomendación condicional. Calidad de la evidencia: baja**
√	La administración de líquidos puede llevar a una sobrecarga de volumen con falla respiratoria, en particular en pacientes con SIRA. Si no hay respuesta a la carga de líquidos o aparecen signos de sobrecarga (distensión venosa yugular, crepitaciones en la auscultación del pulmón, edema pulmonar en los estudios por imágenes o hepatomegalia) se debe reducir o suspender la administración de líquidos. **Punto de buena práctica**
26	En la reanimación aguda de pacientes adultos con COVID-19 y en estado de choque, se recomienda no administrar almidones de hidroxietilo, gelatinas o dextranos. **Recomendación fuerte. Calidad de la evidencia: baja**
27	En la reanimación aguda de pacientes adultos con COVID-19 y en estado de choque, se sugiere no administrar albúmina de forma sistemática para la reanimación inicial. **Recomendación condicional. Calidad de la evidencia: baja**

**Pregunta 7. ¿Cuál es la efectividad y seguridad de los vasopresores y corticoesteroides para el tratamiento de pacientes críticos con COVID-19 en estado de choque?**	
**N.°**	**Recomendación**
28	En pacientes adultos con COVID-19 y en estado de choque, se sugiere administrar norepinefrina como agente vasoactivo de primera línea en lugar de otros agentes. **Recomendación condicional. Calidad de la evidencia: baja**
29	En pacientes adultos con COVID-19 y en estado de choque, si no se dispone de norepinefrina, se sugiere administrar vasopresina o epinefrina, de acuerdo con la disponibilidad, como agente vasoactivo de primera línea en lugar de otros agentes vasoactivos. **Recomendación condicional. Calidad de la evidencia: baja**
30	En pacientes adultos con COVID-19 y en estado de choque, se recomienda no administrar dopamina, dado su bajo perfil de seguridad comparado con los otros vasopresores. **Recomendación fuerte. Calidad de la evidencia: moderada**
31	En pacientes adultos con COVID-19 y en estado de choque, se sugiere valorar los agentes vasoactivos para alcanzar una presión arterial media (PAM) de entre 60 y 65 mmHg, en lugar de una PAM más alta. **Recomendación condicional. Calidad de la evidencia: baja**
32	En pacientes adultos con COVID-19 y en estado de choque, si la PAM prevista no puede alcanzarse mediante la norepinefrina, se sugiere adicionar vasopresina como agente de segunda línea cuando se requiere asociar vasopresores.. **Recomendación condicional. Calidad de la evidencia: moderada**
33	En adultos con COVID-19 y en estado de choque con señales de insuficiencia cardíaca e hipoperfusión persistente posterior a la reanimación con líquidos y la norepinefrina, se sugiere adicionar dobutamina (realizando ecocardiografía previa) en lugar de aumentar la dosis de norepinefrina. **Recomendación condicional. Calidad de la evidencia: muy baja**
34	En pacientes adultos con COVID-19 y en estado de choque que requieren la adición de un segundo vasopresor, se sugiere administrar dosis bajas de corticoesteroides. **Recomendación condicional. Calidad de la evidencia: baja**
√	Se deben administrar vasopresores a los pacientes con COVID-19 cuando el estado de choque persiste durante o después de la reanimación con líquidos hasta alcanzar la PAM establecida y mejoría de los marcadores de perfusión. Si los catéteres venosos centrales (CVC) no están disponibles, los vasopresores pueden ser administrados a través de un catéter intravascular periférico (por un tiempo corto, a dosis bajas) monitoreado cercanamente por signos de extravasación y necrosis, mientras se logra la colocación del CVC. Debe intentarse pasar a un CVC en las primeras 24-48 horas del uso de vasopresores. **Punto de buena práctica**

**Pregunta 8. ¿Cuál es la utilidad de los estudios por imágenes para orientar el tratamiento de los pacientes críticos con COVID-19?**	
**N.°**	**Recomendación**
35	Para pacientes hospitalizados con síntomas graves (riesgo alto de progresión de la enfermedad, no responden al tratamiento de oxígeno suplementario o presentan sospecha clínica de fibrosis pulmonar, tromboembolismo pulmonar o trombosis coronaria), se sugiere tomar imágenes diagnósticas para orientar el manejo terapéutico, además de la evaluación clínica y de laboratorio. **Recomendación condicional. Calidad de la evidencia: muy baja**
√	Se debe seleccionar la modalidad diagnóstica con base en la disponibilidad, la localización del deterioro presentado, el tipo de paciente (p. ej., en ventilación mecánica) y el diagnóstico que se busca establecer. Se sugiere utilizar, de preferencia, la tomografía computarizada o radiografía de tórax o ecografía de pulmón. **Punto de buena práctica**

**Pregunta 9. ¿Cuál es la eficacia y seguridad de las intervenciones farmacológicas para el tratamiento de los pacientes críticos con COVID-19 en la unidad de cuidados intensivos?**	
**N.°**	**Recomendación**
36	No se recomienda la administración de remdesivir, lopinavir y ritonavir, cloroquina e hidroxicloroquina con o sin azitromicina, colchicina y plasma convaleciente para el manejo de los pacientes con COVID-19, ni para realizar ensayos clínicos. **Recomendación fuerte. Calidad de la evidencia: baja y moderada**
37	Se sugiere el uso de tocilizumab en pacientes críticos que ingresaron en la UCI debido a una descompensación respiratoria rápida. *Se debe administrar una dosis intravenosa única de tocilizumab (8 mg/kg de peso hasta 800 mg) en combinación con corticoesteroides (p. ej., dexametasona en dosis de 6 mg/día por 10 días) en las primeras 72 horas de hospitalización o ingreso a UCI, según la presencia de marcadores de inflamación. Esta recomendación no aplica a los pacientes que recibieron tocilizumab en estadio grave de la enfermedad. **Recomendación condicional. Calidad de la evidencia: moderada**
38	No se sugiere la administración de antiparasitarios, antivirales, N-acetil cisteína ni inmunomoduladores (excepto tocilizumab), fuera del contexto de ensayos clínicos. **Recomendación condicional. Calidad de la evidencia: baja**
39	Se recomienda administrar corticoesteroides en dosis bajas a los pacientes críticos que reciben oxígeno suplementario o se encuentran ventilados, con el fin de disminuir la mortalidad y la progresión a ventilación mecánica invasiva **Recomendación fuerte. Calidad de la evidencia: moderada**
40	En pacientes adultos con COVID-19 en ventilación mecánica, se sugiere utilizar agentes antimicrobianos o antibacterianos de forma empírica durante 5 a 7 días ajustados a protocolos institucionales y sobre la base del diagnóstico clínico (p. ej., neumonía adquirida en la comunidad, sepsis o sospecha de infección bacteriana asociada) y los datos locales de resistencia bacteriana. **Recomendación condicional. Calidad de la evidencia: baja**
√	La administración de antibióticos debe iniciarse en menos de una hora de la evaluación del paciente. La terapia antibiótica debe ser ajustada con base en los resultados microbiológicos y el criterio clínico. **Punto de buena práctica**
√	El tratamiento de coinfecciones debe ser realizado con base en la confirmación diagnóstica y el criterio clínico siguiendo los protocolos institucionales **Punto de buena práctica**
41	En pacientes adultos con COVID-19 que desarrollen fiebre, se sugiere utilizar medicamentos para el control de la temperatura. Su selección depende de la comorbilidad de cada paciente. **Recomendación condicional. Calidad de la evidencia: baja**
42	Se sugiere no administrar antiinflamatorios no inflamatorios a los pacientes críticos con COVID-19, con el fin de disminuir las complicaciones pleuropulmonares. **Recomendación condicional. Calidad de la evidencia: muy baja**

**Pregunta 10. ¿cuáles son los lineamientos para la prevención de complicaciones asociadas al tratamiento de los pacientes críticos con COVID-19?**	
**N.°**	**Recomendación**
43	En pacientes críticos sin contraindicación para recibir anticoagulantes, se recomienda usar profilaxis farmacológica con heparina de bajo peso molecular (HBPM), de acuerdo con los estándares locales e internacionales, para prevenir el tromboembolismo venoso. En los pacientes con contraindicaciones, se sugiere utilizar profilaxis mecánica (dispositivo de compresión neumática intermitente). **Recomendación fuerte. Calidad de la evidencia: muy baja**
√	Se sugiere que se identifique los pacientes con alto riesgo de tromboembolismo de acuerdo con los siguientes marcadores: niveles elevados de proteína C reactiva, fibrinógeno y dímero D. Los pacientes críticos con COVID-19 y alto riesgo de tromboembolismo, sin complicaciones renales y con bajo riesgo de sangrado, deben recibir 1 mg/kg por día de enoxaparina durante por lo menos 7 días. Se debe realizar seguimiento de efectos secundarios y marcadores pronósticos para ajustar la dosis de enoxaparina a 40 mg. **Punto de buena práctica**
√	La terapia profiláctica debe comenzar dentro de las primeras 14 horas de la admisión y continuarla por 7 días o lo que dure la estadía hospitalaria. Si los pacientes están recibiendo anticoagulación al momento del ingreso a la UCI, deben continuar con el régimen terapéutico establecido. **Punto de buena práctica**
44	En casos seleccionados de pacientes con uso de vasopresores continuado, se sugiere la administración de inhibidores de la bomba de protones a dosis profiláctica por períodos cortos, para evitar el sangrado de úlceras por estrés. Se debe vigilar a los pacientes para controlar el riesgo de ocurrencia de infecciones asociadas a la atención en salud. **Recomendación condicional. Calidad de la evidencia: muy baja**
√	Se deben considerar con cuidado las interacciones medicamentosas y los efectos secundarios de los medicamentos administrados que puedan afectar la sintomatología de COVID-19 (incluidos los efectos en las funciones respiratoria, cardíaca, neurológica, mental e inmunitaria). **Punto de buena práctica**
√	Se recomienda implementar las siguientes intervenciones con el fin de prevenir complicaciones asociadas al manejo de pacientes críticos con COVID-19: Reducir la incidencia de neumonía asociada al ventilador Usar un protocolo institucional de destete del ventilador que incluya la evaluación diaria. Se prefiere la intubación oral a la intubación nasal en adolescentes y adultos. Mantener al paciente en posición semisentada (cabeza con elevación de 30° a 45°). Utilizar un circuito cerrado de aspiración, drenar y eliminar el condensado en las tubuladuras en forma periódica. Utilizar un circuito nuevo para cada paciente; una vez que el paciente esté ventilado, no cambiar el circuito de manera sistemática, sino solo si está sucio o dañado. Cambiar el intercambiador de calor cuando no funcione correctamente, cuando esté sucio o cada 5 a 7 días. Reducir la incidencia de infecciones sanguíneas asociadas a dispositivos intravasculares Utilizar una lista de verificación como recordatorio de cada paso necesario para la inserción estéril y como recordatorio diario para quitar el dispositivo intravascular si ya no es necesario. Reducir la incidencia de úlceras por presión Lateralizar al paciente cada dos horas. Movilizar en forma activa al paciente en el momento de la enfermedad que sea seguro para hacerlo. Reducir la incidencia de úlceras por estrés y sangrado gastrointestinal Dar nutrición enteral temprana (dentro de las 24 a 48 horas del ingreso). **Punto de buena práctica**
√	Es clave identificar y manejar las posibles causas subyacentes del síndrome confusional (a menudo multicausales), y realizar una evaluación periódica de los factores de riesgo, proporcionar una movilización y reorientación tempranas del paciente, promover la normalización del ciclo de sueño-vigilia, asegurar una comunicación eficaz y brindar tranquilidad al paciente, involucrando a familiares y cuidadores por vía virtual. **Punto de buena práctica**
45	Se sugiere administrar haloperidol en dosis bajas (0,5 mg, hasta un máximo de 10 mg/día) inicialmente a los pacientes en UCI con síndrome confusional que no han respondido a intervenciones no farmacológicas para el manejo del síndrome (reorientación, calendarios, relojes, iluminación natural, reducción del ruido ambiental, favorecer el sueño y evitar fármacos que causen síndrome confusional, entre otros). **Recomendación fuerte. Calidad de la evidencia: muy baja**
46	Se sugiere monitorear y manejar a los pacientes críticos por las siguientes manifestaciones neurológicas y cardíacas: dolor de cabeza, confusión, alteraciones de la conciencia, síntomas del sistema nervioso periférico, evento cerebrovascular y epilepsia. **Recomendación condicional. Calidad de la evidencia: muy baja**

**Pregunta 11. ¿Cuál es la eficacia y seguridad de la rehabilitación temprana en los pacientes con COVID-19 en la unidad de cuidado intensivo?**	
**N.°**	**Recomendación**
47	Para los pacientes con COVID-19 hospitalizados en la UCI, se sugiere realizar rehabilitación temprana con el objetivo de disminuir la debilidad adquirida en la UCI. Recomendación condicional. Calidad de la evidencia: muy baja
√	El tipo de rehabilitación temprana depende del paciente, el tipo de ventilación, si se encuentra con sedación y los recursos disponibles por la institución. **Punto de buena práctica**

**Pregunta 12. ¿Cuáles son los criterios de egreso de los pacientes con COVID-19 en la unidad de cuidado intensivo?**	
**N.°**	**Recomendación**
48	Para los pacientes con COVID-19 hospitalizados en la UCI, cuyos síntomas han mejorado, se sugiere realizar una evaluación clínica y de laboratorio, y verificar que no se requiere soporte respiratorio, renal o hemodinámico, para tomar la decisión de egreso de la unidad. **Recomendación condicional. Calidad de la evidencia: muy baja**
49	Para los pacientes que reciben anticoagulantes orales antes del ingreso a la UCI, se recomienda estratificar el riesgo de presentar tromboembolismo venoso después del egreso y considerar extender la profilaxis con la administración de una dosis estándar. **Recomendación condicional. Calidad de la evidencia: muy baja**
√	Para los pacientes que han sido dados de alta de la UCI intensivo, se recomienda evaluar la capacidad de deglución, la movilidad, la presencia de síndrome confusional, el deterioro cognitivo y la salud mental. Con base en la evaluación, se determinan los requerimientos de rehabilitación y seguimiento. **Punto de buena práctica**
50	Se sugiere que los pacientes que cumplen los criterios de egreso de la UCI salgan con un plan de salida que incluya un resumen del diagnóstico al egreso, medicamentos y plan de cuidado; así como proveer información a la familia y al paciente sobre su cuidado. **Recomendación Condicional. Calidad de la evidencia: muy baja**
√	Debe realizarse un programa de rehabilitación desde la salida de la UCI hasta el largo plazo; con derivación a los servicios o centros especializados de rehabilitación designados para atender los pacientes con COVID-19 que siguen infecciosos. Considerar la posibilidad de realizar las actividades programadas de forma virtual. **Punto de buena práctica**
√	Los programas de rehabilitación deben ser ejecutados por equipos multidisciplinarios y deben estar orientados a las necesidades y metas de los pacientes, que incluyen terapia física; educación y consejo en estrategias de autocuidado; técnicas respiratorias; apoyo a cuidadores; grupos de apoyo; manejo de estrés y modificaciones en el hogar. **Punto de buena práctica**

**Pregunta 13. ¿Cuáles son las indicaciones de diálisis temprana en los pacientes con COVID-19 y daño renal en la unidad de cuidado intensivo?**	
**N.°**	**Recomendación**
√	En los pacientes con SIRA por COVID-19 que desarrollen lesión renal aguda, se sugiere utilizar hemodiálisis, según la disponibilidad, en caso de presentar criterios agudos de diálisis, y optimizar el balance hídrico. **Punto de buena práctica**

**CUADRO 4.** Barreras, facilitadores y estrategias de implementación relacionados con manejo de pacientes críticos con COVID-19

**Aspecto**	**Barreras**	**Facilitadores**	**Estrategias de implementación**
Recurso humano	Los países de la Región no cuentan con suficiente personal capacitado para desarrollar las recomendaciones de cuidado de los pacientes críticos con COVID-19. En algunos países de la Región no existe currículo profesional en el nivel nacional para la formación de recurso humano en los niveles de prestación de servicios, incluidas las UCI. Los trabajadores de la salud se sienten sobrecargados y consideran el uso del equipo de protección como una tarea adicional, no reciben entrenamiento adecuado para su uso correcto, no cuentan con cuartos de aislamiento y no cuentan con suficientes equipos de protección personal (EPP).	Proveedores de servicios de salud, sociedades científicas, instituciones académicas y entidades gubernamentales, incluido el ministerio de salud	Capacitar a los profesionales de la salud y al personal en formación sobre el manejo clínico de pacientes con COVID-19, así como sobre las recomendaciones de prevención y control de infecciones (o uso racional del EPP) considerando la evidencia más actualizada. Proveer supervisión y retroalimentación continua de verificación de implementación de la guía mediante una metodología estandarizada. El manejo de los pacientes con COVID-19 debe realizarse en un ambiente seguro y los profesionales de la salud deben contar con el EPP recomendado para la contención de patógenos emergentes en los establecimientos de salud, los entornos hospitalarios y entre los familiares (o la comunidad). Socializar los algoritmos y las recomendaciones clave de la guía.
Conocimiento de la guía	Los profesionales de la salud no tienen acceso a la guía. Los profesionales de la salud no conocen que existe una guía o no conocen las recomendaciones disponibles. Los profesionales de la salud reciben presión por parte de pacientes y la sociedad para utilizar tecnologías cuya eficacia y seguridad no han sido probadas en ensayos clínicos de calidad adecuada.	Proveedores de servicios de salud Entidades gubernamentales Sociedades científicas Instituciones académicas	Socializar la guía con los profesionales de la salud para que sepan dónde encontrarla en los proveedores de salud. Involucrar a los líderes de opinión e identificar promotores de la implementación de las recomendaciones. Publicar los algoritmos y la guía en páginas web o intranet de las instituciones, aplicaciones móviles, boletines electrónicos institucionales o páginas especializadas como apoyo al proceso de consulta rápida. Las recomendaciones clave para implementar en cada institución se muestran al abrir la historia clínica a través de medios electrónicos.
Insumos	No todos los establecimientos de salud cuentan con EPP en cantidades adecuadas y que cumplan con los estándares internacionales para todos los profesionales de la UCI. La identificación de los marcadores pronósticos mediante pruebas de laboratorio puede implicar un aumento en los costos para el manejo de los pacientes en varios establecimientos de salud de la Región de las Américas. Algunos establecimientos de salud, especialmente en áreas remotas, no cuentan con estudios por imágenes diagnósticos, pulsímetros, oxígeno, ventiladores y terapias recomendadas En una gran cantidad de UCI de la Región no se cuenta con cuartos con presión negativa como parte de los controles en prevención y control de infecciones en el contexto de la pandemia por COVID-19. Los establecimientos de salud pueden no contar con las condiciones estructurales mínimas para proveer cuidados de la salud como agua y saneamiento, equipos y dispositivos médicos como EPP y medicamentos, ni espacio físico adecuado y suficiente para aislar casos sospechosos o confirmados.	Entidades gubernamentales Proveedores de servicios de salud Distribuidores de medicamentos	Los sistemas de salud, establecimientos de salud o instituciones prestadoras de servicios de salud necesitan asegurar que los insumos necesarios estén disponibles. Esto incluye el fortalecimiento de procesos regulatorios y logísticos. La OPS ha desarrollado documentos técnicos de apoyo para los procesos regulatorios y técnicos del uso de oxímetros, uso de ventilación natural para el control de infecciones, y uso de zonas de triaje, los cuales pueden ser usados para fortalecer la adquisición de los insumos necesarios. Se debe aumentar la capacidad instalada de los países para lograr una mayor provisión de los insumos necesarios a todas las instituciones que lo necesitan para brindar el soporte necesario a los pacientes críticos con COVID-19. Fortalecer la generación de políticas informadas en la evidencia de los países. Invitar a los médicos y a los tomadores de decisiones a conocer los beneficios, los riesgos y los costos de las intervenciones farmacológicas a administrar en los pacientes críticos con COVID-19.
Acceso	En zonas remotas se cuenta con poco acceso a UCI y a especialistas capacitados. Se presentan dificultades en la cadena de provisión de oxígeno. Se pueden presentar dificultades de acceso, dado que se evidencian diferencias en los países de la Región y dentro de cada país para la provisión de oxígeno. En varios países de la Región, el oxígeno solo se encuentra disponible en hospitales urbanos y en proveedores privados de servicios de salud, lo cual hace que su acceso no sea equitativo. Bajo suministro de medicamentos esenciales para el manejo de pacientes con COVID-19. Algunos medicamentos recomendados pueden presentar un costo elevado, que disminuye su acceso.	Entidades gubernamentales Proveedores de servicios de salud Distribuidores de medicamentos	Se debe contar con mecanismos de compra y distribución controlados y rápidos de los insumos necesarios para el manejo adecuado de los pacientes críticos con COVID-19 Fortalecer las normas técnicas, los programas nacionales y las políticas que buscan apoyar la implementación de guías de práctica clínica y mejorar el acceso a los servicios de salud de los pacientes con COVID-19. Fomentar estrategias que permitan aumentar la velocidad de producción y provisión de oxígeno en los países, así como fortalecer la gestión coordinada de la cadena mundial de suministro mediante las alianzas con organismos nacionales e internacionales. Fortalecer la adquisición de medicamentos con el apoyo de mecanismos internacionales y negociar de los precios de medicamentos.

**FIGURA 2.**
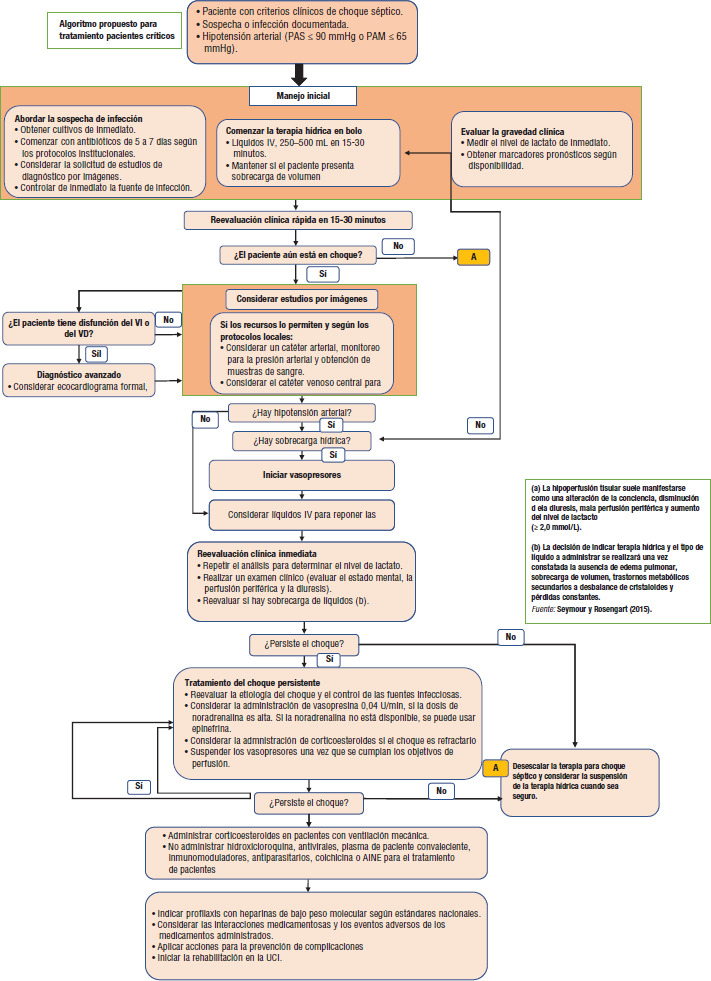
Algoritmo propuesto para el tratamiento de pacientes críticos con choque séptico

**FIGURA 3.**
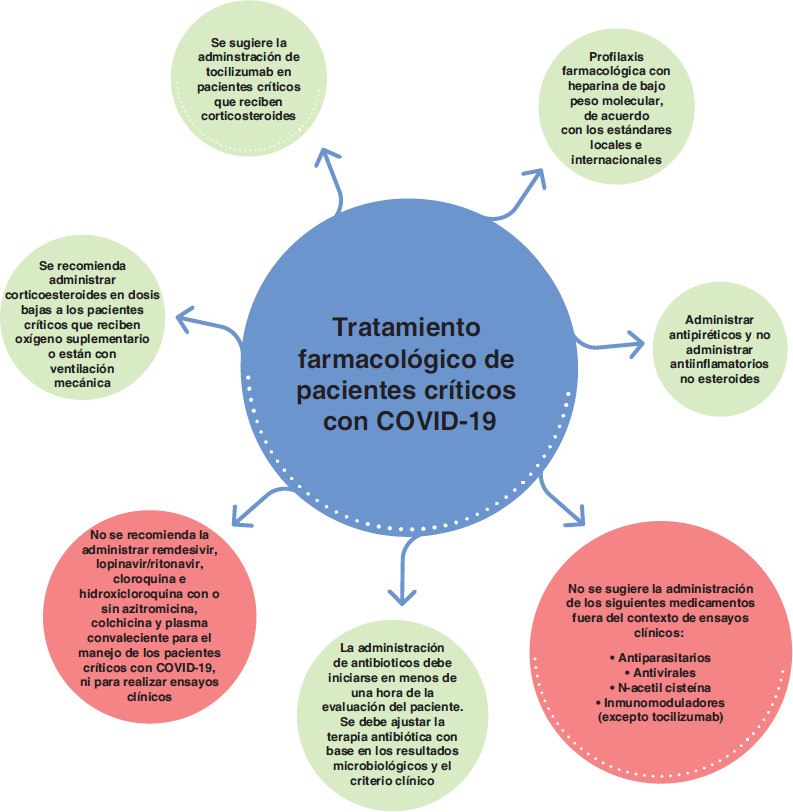
Recomendaciones de tratamiento farmacológico para el manejo de pacientes críticos con COVID-19

**CUADRO 5.** Indicadores de proceso y resultado en la implementación de la guía para el cuidado de pacientes críticos con COVID-19

**Indicadores de proceso**
Proporción de pacientes críticos con COVID-19 con ventilación mecánica que recibieron tratamiento farmacológico durante la estancia en la UCI.
Proporción de pacientes con COVID-19 con SIRA y dificultad respiratoria, hipoxemia o choque (sin intubación o ventilación mecánica) que recibieron oxígeno suplementario hasta alcanzar SpO2 ≥94%.
Proporción de pacientes que recibieron un plan de salida con rehabilitación al momento del egreso hospitalario.

**Indicadores de resultado**
Tasas de mortalidad por COVID-19.
Número de pacientes en UCI.
Número de pacientes con ventiladores debido a COVID-19 y sus complicaciones.

UCI, unidad de cuidado intensivo; SIRA, síndrome de infección respiratoria aguda; SpO_2_, saturación parcial de oxígeno.

## CONCLUSIONES

La Organización Panamericana de la Salud pone a disposición de los gestores y del personal de la salud una síntesis sobre las recomendaciones informadas en la evidencia para el cuidado de pacientes críticos con COVID-19, con base en la evidencia más actualizada que provee mayor beneficio a los pacientes. Asimismo, presenta aspectos a considerar, como algunas barreras para la implementación de las recomendaciones y estrategias para abordarlas, así como indicadores de proceso y resultado. Esperamos que esta síntesis favorezca la diseminación y el uso de las guías que elabora la OPS y contribuya a mejorar la calidad de la atención de los pacientes durante la pandemia.

## References

[1] 1. Organización Mundial de la Salud. Coronavirus disease (COVID-19) outbreak. Ginebra: OMS; 2020. Disponible en: https://www.who.int/emergencies/diseases/novel-coronavirus-2019

[2] 2. Zhou F, Yu T, Du R, Fan G, Liu Y, Liu Z, et al. Clinical course and risk factors for mortality of adult inpatients with COVID-19 in Wuhan, China: a retrospective cohort study. The Lancet. 2020;395(10229):1054-1062. Disponible en: 10.1016/S0140-6736(20)30566-3 PMC7270627 32171076

[3] 3. Organización Mundial de la Salud. Clinical care of severe acute respiratory infections – Tool kit. COVID-19 adaptation. Ginebra : OMS ; 2020. Disponible en: https://www.who.int/publications/i/item/clinical-care-of-severe-acute-respiratory-infections-tool-kit

[4] 4. Organización Mundial de la Salud. Clinical management of severe acute respiratory infection when COVID-19 is suspected. Ginebra: OMS; 2020. Disponible en: https://www.who.int/publications-detail/clinical-management-of-severe-acute-respiratory-infection-when-novel-coronavirus-(ncov)-infection-is-suspected. Fecha de acceso: julio del 2020.

[5] 5. Parohan M, Yaghoubi S, Seraji A, Javanbakht MH, Sarraf P, Djalali M. Risk factors for mortality in patients with Coronavirus disease 2019 (COVID-19) infection: a systematic review and meta-analysis of observational studies. The Aging Male. 2020;23(5):1416-1424. Disponible en: 10.1080/13685538.2020.1774748 32508193

[6] 6. Public health emergency SOLIDARITY trial of treatments for COVID-19 infection in hospitalized patients. ISRCTN identifier: 83971151. 2020. Acceso el 1 de julio de 2020. Disponible en: http://www.isrctn.com/ISRCTN83971151

[7] 7. RECOVERY Collaborative Group, Horby P, Lim WS, et al. Dexamethasone in hospitalized patients with COVID-19: preliminary reportt [published online ahead of print, 2020 Jul 17]. N Engl J Med. 2021; 384:693-704. Disponible en: doi:10.1056/NEJMoa2021436 10.1056/NEJMoa2021436 PMC7383595 32678530

[8] 8. Organización Panamericana de la Salud. Guía para el cuidado crítico de pacientes adultos graves con Coronavirus (COVID-19) en las Américas (Versión 3.0). Washington D.C.: OPS; 2021. Disponible en: https://iris.paho.org/handle/10665.2/52529 (Para el autor: este enlace lleva a la versión 2 del documento).

[9] 9. Organización Mundial de la Salud. Handbook for Guideline Development (2nd ed.). Ginebra: OMS; 2014. Disponible en: https://www.who.int/publications/guidelines/handbook_2nd_ed.pdf?ua=1 Acceso en junio del 2016.

[10] 10. Guyatt GH, Oxman AD, Kunz R, Atkins D, Brozek J, Vist G, et al. GRADE guidelines: 2. Framing the question and deciding on important outcomes. J Clin Epidemiol. 2011;64(4):395-400. 10.1016/j.jclinepi.2010.09.012 21194891

[11] 11. DECIDE Grade 2011-2015. Evidence to Decision (EtD) Framework. Disponible en: http://www.decide-collaboration.eu/evidence-decision-etd-framework). Acceso en agosto del 2019.

[12] 12. Organización Panamericana de la Salud. Algoritmo de manejo de pacientes con sospecha de infección por COVID-19 en el primer nivel de atención y en zonas remotas de la Región de las Américas, julio del 2020. Washington D.C.: OPS; 2020. Disponible en: https://iris.paho.org/handle/10665.2/52501

[13] 13. Lewis CC, Fischer S, Weiner BJ, et al. Outcomes for implementation science: an enhanced systematic review of instruments using evidence-based rating criteria. Implementation Sci. 2015;10:155. 10.1186/s13012-015-0342-x PMC4634818 26537706

[14] 14. Houghton C, Meskell P, Delaney H, Smalle M, Glenton C, Booth A, et al. Barriers and facilitators to healthcare workers' adherence with infection prevention and control (IPC) guidelines for respiratory infectious diseases: a rapid qualitative evidence synthesis. The Cochrane database of systematic reviews. 2020;4(4):CD013582. Disponible en: 10.1002/14651858.CD013582 PMC7173761 32315451

[15] 15. Organización Panamericana de la Salud. Aspectos técnicos y regulatorios sobre el uso de oxímetros de pulso en el monitoreo de pacientes con COVID-19, 4 de agosto del 2020. Washington D.C.: OPS; 2020. Disponible en: https://iris.paho.org/handle/10665.2/52551

[16] 16. Organización Panamericana de la Salud. Triage experience in a global pandemic: setting up triaje stations (or similar) for surge capacity and a possible strategy for mechanical ventilator shortage. Rapid review. Washington D.C.: OPS; 2020. En proceso de publicación.

[17] 17. Organización Panamericana de la Salud. Ventilación natural para el control de las infecciones en entornos de asistencia sanitaria. Washington D.C.: OPS; 2010. Disponible en: https://www.paho.org/hq/dmdocuments/2011/ventilacion_natual_spa_25mar11.pdf. Acceso en marzo del 2020.

[18] 18. Banco Mundial. Oxygen for all, during COVID-19 (coronavirus) and beyond. Washington D.C.: Bando Mundial; 20202. Disponible en: https://blogs.worldbank.org/health/oxygen-all-during-covid-19-coronavirus-and-beyond. Acceso en junio del 2020.

[19] 19. Stein F, Perry M, Banda G, Woolhouse M, Mutapi F. Oxygen provision to fight COVID-19 in sub-Saharan Africa. BMJ Global Health: 2020;5(6):e002786. Disponible en: 10.1136/bmjgh-2020-002786 PMC7295423 32532759

